# Histological, physiological, and transcriptomic responses of hepatopancreas to air exposure in asian freshwater clam *Corbicula fluminea*

**DOI:** 10.3389/fphys.2022.952744

**Published:** 2022-08-11

**Authors:** Ting Zhang, Dongpu Xu, Guohua Lv, Anqi Wang, Haibo Wen

**Affiliations:** Key Laboratory of Freshwater Fisheries and Germplasm Resources Utilization, Ministry of Agriculture and Rural Affairs, Freshwater Fisheries Research Center, Chinese Academy of Fishery Sciences, Wuxi, China

**Keywords:** *C. fluminea*, air exposure, histology, physiology, transcriptome

## Abstract

*Corbicula fluminea* (*C. fluminea*) is an important freshwater economy shellfish in China, but it often suffers from air exposure during transportation. In this study, we investigated the histological, physiological (mainly including respiratory metabolism, antioxidant capacity, and immune function), and transcriptomic responses of hepatopancreas in *C. fluminea* to different times of air exposure. At histological level, air exposure caused vacuolation of digestive cells (24–96 h) and enlargement of digestive tubule lumen (6–96 h) in hepatopancreas. At physiological level, the activities of enzymes related to glycolysis (hexokinase and pyruvate kinase) and anaerobic respiration (lactate dehydrogenase) were increased first (6–24 h) of air exposure, then came back to normal level or even decreased. The activity of aerobic respiration-related enzyme (succinic dehydrogenase) began to reduce from 24 h of air exposure. The activities of antioxidant enzymes (superoxide dismutase and catalase) were enhanced during 6–48 h of air exposure and then returned to control level or even inhibited. The content of malondialdehyde (MDA) increased from 96 h of air exposure. The activities of immune-related enzymes (acid phosphatase and alkaline phosphatase) increased during 6–48 h, then returned to normal or began to decline. At transcriptome level, 44 differentially expressed genes (DEGs) in the hepatopancreas were identified after 96-h air exposure. Among these DEGs, 8 were associated with glycolysis, TCA cycle, immune, and antioxidant, and were downregulated after 96-h air exposure. Taken together, these findings illuminated the response of *C. fluminea* to air exposure at histological, physiological, and transcriptomic levels, which will be beneficial to the aquaculture and transportation of *C. fluminea*.

## Introduction

Aquatic animals often suffer from many stresses during the aquaculture process, which will harm their health and the aquaculture industry. Among many stresses, air exposure is unavoidable stress for most aquatic animals and may occur during ebb tide, harvest, and transportation (Yin et al., 2017; Zhou et al., 2021). When exposed to air, aquatic animals have less access to oxygen and face severe stress of hypoxia. Lots of evidence indicate that severe air exposure will cause several adverse effects on aquatic animals, such as oxidative stress injury, cell apoptosis, destruction of the histological structure, disruption of energy homeostasis, poor muscle quality, reduced growth, and even death (Arlinghaus and Hallermann, 2007; Gu et al., 2017; Lu et al., 2021; Harianto et al., 2022). To cope with desiccation and hypoxia stress of air exposure, aquatic animals can mobilize multiple adaptive physiological and molecular mechanisms, including changing respiratory metabolic strategies, enhancing immune function, increasing antioxidant defense, and altering gene expression (Romero et al., 2007; Bao et al., 2019). At present, there are many studies on the response of various aquatic animals, including shellfish, to air exposure. Whereas most studies on shellfish mainly focus on intertidal marine shellfish (Meng et al., 2018; Nie et al., 2020), there are a few studies on freshwater shellfish.

The freshwater shellfish Asian clam *Corbicula fluminea* (*C. fluminea*), which belongs to the class Bivalvia, the family Corbiculidae, and the genus Corbicula, is distributed in many rivers, estuaries, and lakes in China (Rosa et al., 2011). It is an important part of freshwater fisheries resources in China due to its excellent product performance and high economic value (Abaychi and Mustafa, 1988). In recent years, *C. fluminea* and its processing products are selling well in Japan and Southeast Asia countries, making it become one of the important export aquatic products in China (Liao et al., 2013). However, it takes a long time to transport *C. fluminea* from harvest to market. To reduce transport costs, the transportation of *C. fluminea* is usually waterless, which makes *C. fluminea* face the risk of air exposure. Therefore, it is necessary to clarify the responses of *C. fluminea* to air exposure for their aquaculture and transportation. Although the previous study has elucidated the behavior and metabolic response of *C. fluminea* to air exposure (Byrne et al., 1990), the histological, physiological, and molecular responses have been poorly studied.

In this study, we investigated the effects of air exposure on the histology, physiology (mainly including respiratory metabolism, antioxidant capacity, and immune function) as well as transcriptome of the hepatopancreas of *C. fluminea* for the first time. This study will provide valuable data to clarify the coping mechanism of *C. fluminea* to air exposure and be beneficial to the aquaculture and transportation of *C. fluminea*.

## Materials and methods

### Animals

*C. fluminea* (wet weight: 4.10 ± 0.90 g; shell length: 25.32 ± 2.02 mm; shell height: 20.88 ± 1.99 mm; shell width: 13.37 ± 1.24 mm) were obtained from the Anhui Shuiyun Environmental Protection Technology Co., LTD. (China, Wuhu). Before exposure experiments, all *C. fluminea* were kept in dechlorinated freshwater with a temperature of 20°C ± 0.5°C, dissolved oxygen of 6.16 ± 0.13 mg/L, and a pH of 8.3 ± 0.1 for 2 weeks of acclimation. In this period, clams were fed twice daily with chlorella. All animal procedures followed the Animal Experiments Ethics Committee of Freshwater Fisheries Research Center, Chinese Academy of Fishery Sciences.

### Air exposure and sampling

The *C. fluminea* were randomly divided into seven groups: 0, 6, 12, 24, 48, 72, and 96 h. Three replications were set up in each group. Clams in the 0 h group were used as controls and reared as they had been during acclimation. The individuals of other groups were placed into aquariums without water to undergo the indicated times of air exposure. The hepatopancreas tissues of clams in each group were collected for further analysis.

### Histopathological analysis

The hepatopancreas of four clams from each group was fixed in Bouin’s fixative solution (Phygene, China). After being dehydrated and transparentized, tissues were embedded in paraffin wax and cut into 6-μm-thick sections. Then, the paraffin sections were stained with hematoxylin/eosin (H & E) and observed under a microscope (Olympus, Japan) (Mi et al., 2021).

### Biochemical detection

The hepatopancreas tissues were homogenized with 0.9% saline solution at a ratio of 1:9 on ice and centrifuged at 936 × g at 4°C for 10 min. The supernatants were collected in new tubes to detect the activities of lactate dehydrogenase (LDH), succinate dehydrogenase (SDH), hexokinase (HK), pyruvate kinase (PK), superoxide dismutase (SOD), catalase (CAT), acid phosphatase (ACP) and alkaline phosphatase (AKP), and the contents of glutathione (GSH) and malonaldehyde (MDA). All detection procedures followed the manufacturer’s instructions for commercial assay kits (LDH: A076-1-1; SDH: A022-1-1; HK: A077-3-1; PK: A076-1-1; SOD: A001-3-2; CAT: A007-1-1; ACP: A060-2-2; AKP: A059-2-2; GSH: A006-2-1; MDA: A003-1-2) (Nanjing Jiancheng Bioengineering Institute, China). The protein contents were measured using a Bradford Protein Assay Kit (Beyotime, China). The enzyme activity was normalized by protein concentration.

### RNA extraction, cDNA library construction, and sequencing

The RNA samples of the hepatopancreas tissues in the 0 h group (control: CL) and 96 h group (air exposure: AE) were used for the Illumina transcriptome sequence. The hepatopancreas tissues of 3 clams were mixed into one sample, and 3 samples from each group were utilized for transcriptome sequence. High-quantity RNAs were used for the construction of Illumina cDNA libraries. Then 6 cDNA libraries were sequenced on the Illumina HiSeqX10 platform at Magigene (Guangdong, China). Fragments Per Kilobase Million mapped reads (FPKM) method was utilized to calculate the relative expressions of transcripts. The differentially expressed genes (DEGs) were identified by a threshold of false discovery rate (FDR) <0.05 and |log2 (fold change)| >1.

### Real-time quantitative PCR

The RNA-seq results were verified by real-time quantitative PCR (qRT-PCR). RNA was extracted from the hepatopancreas of six clams from each group using TaKaRa MiniBEST Universal RNA (TaKaRa, Japan). The cDNA was synthesized using PrimeScript™ RT reagent Kit with gDNA Eraser (TaKaRa). The qRT-PCR was performed using B Green™ Premix Ex Taq™ (Tli RNaseH Plus) (TaKaRa). The primers used in qRT-PCR were designed by Primer 5.0 software and their sequence information was shown in Supplementary Table S1. The transcription elongation factor 1 alpha (*EF1α*) was utilized as a reference gene.

### Statistical analysis

Data analyses were performed using SPSS 22.0 software. All data were expressed as mean ± standard deviation (SD). One-way ANOVA combined with the LSD post-test was employed to compare the differences among groups. A *p*-value < 0.05 indicated statistical significance.

## Results

### Histopathological changes in hepatopancreas

**FIGURE 1:**
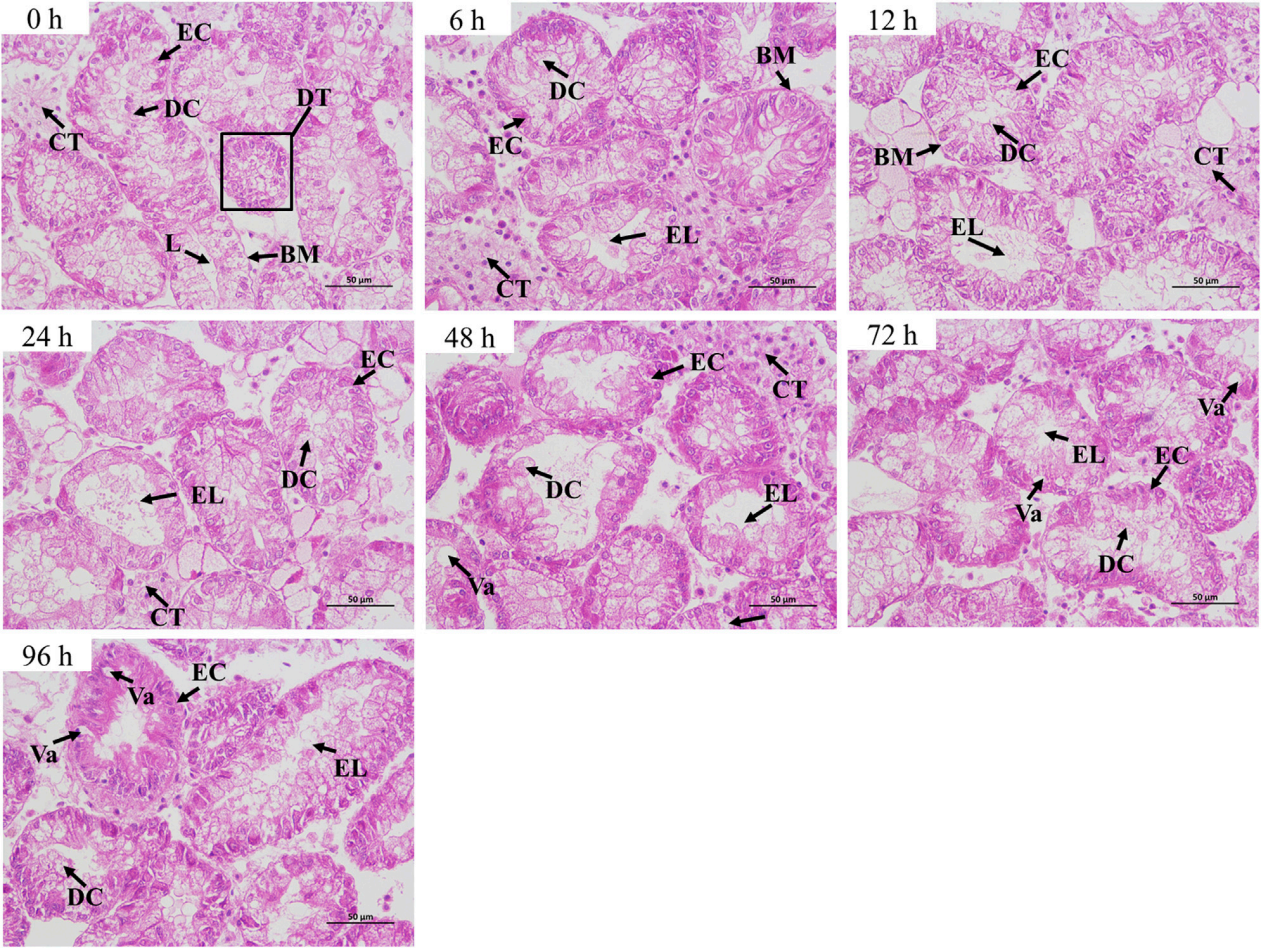
Histopathological changes in the hepatopancreas of *C. fluminea* under air exposure. DT, digestive tubule; L, lumen; EC, epithelial cell; DC, digestive cell; CT, connective tissue; BM, basement membrane; Va, vacuole; EL, enlargement of lumen.

The hepatopancreas of normal *C. fluminea* (0 h group) mainly consisted of the digestive tubule and connective tissue. In the control group, the lumens of the digestive tubule were small or closed and the epithelial cells and digestive cells were in alignment. In air exposure groups, the enlargement of the lumen was observed in the hepatopancreas of *C. fluminea* from 6 to 96 h. After 24 h, the epithelial cells and digestive cells were arranged irregularly and even exfoliated. The vacuolation occurred after 48 h (Figure 1).

### Effects of air exposure on respiratory metabolism

**FIGURE 2:**
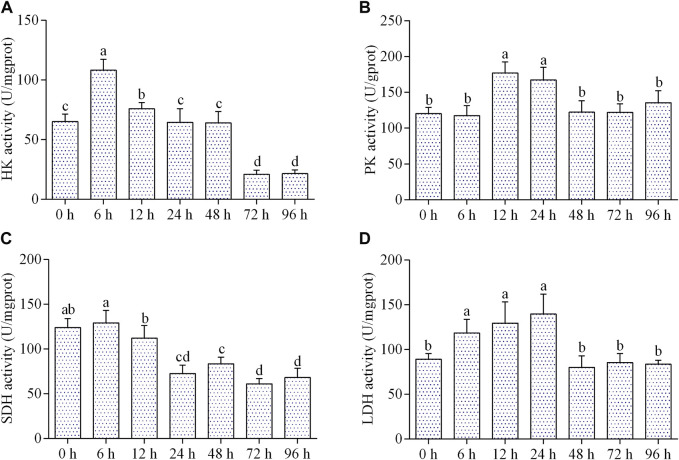
Effects of air exposure on respiratory metabolism. **(A)** HK activity (*n* = 5); **(B)** PK activity (*n* = 5); **(C)** SDH activity (*n* = 5); **(D)** LDH activity (*n* = 5). Data were expressed as mean ± SD. Different letters indicated statistical significance (*p* < 0.05).

The activity of HK increased significantly from 6 to 12 h of air exposure, then kept normal level from 24 to 48 h, and dropped remarkably from 72 to 96 h (Figure 2A). The activity of PK was significantly elevated from 12 to 24 h of air exposure and then came back to the control level during 48–96 h (Figure 2B). As shown in Figure 2C, the activity of SDH significantly decreased from 24 to 96 h after air exposure. LDH activity elevated significantly from 6 to 24 h of air exposure, and then returned to normal level during 48–96 h (Figure 2D).

### Effects of air exposure on antioxidant and oxidative damage parameters

**FIGURE 3:**
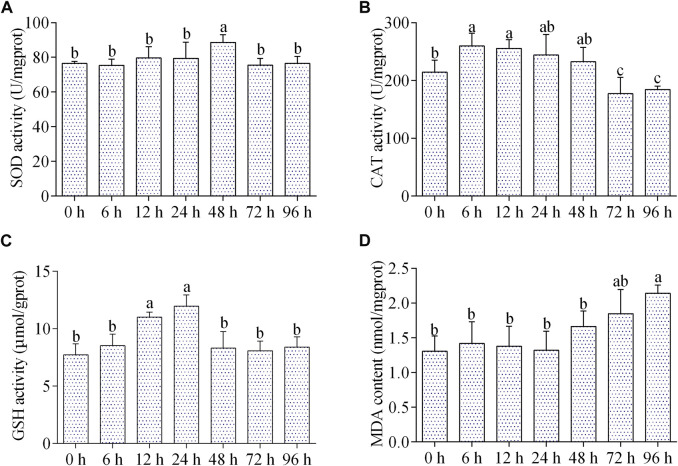
Effects of air exposure on antioxidant parameters in hepatopancreas. **(A)** SOD activity; **(B)** CAT activity; **(C)** GSH content; **(D)** MDA content. Data were expressed as mean ± SD (*n* = 5). Different letters indicated statistical significance (*p* < 0.05).

The SOD activity was significantly increased at 48 h of air exposure, and then returned to the control level from 72 to 96 h (Figure 3A). The CAT activity increased significantly during 6–12 h of air exposure, then returned to normal level from 24 to 48 h, and reduced markedly from 72 to 96 h (Figure 3B). The GSH content was increased between 12–24 h, and then come back to normal (Figure 3C). The content of MDA was remarkably increased after 96 h of air exposure, while did not change significantly at other time points (Figure 3D).

### Effects of air exposure on immune enzyme activities

**FIGURE 4:**
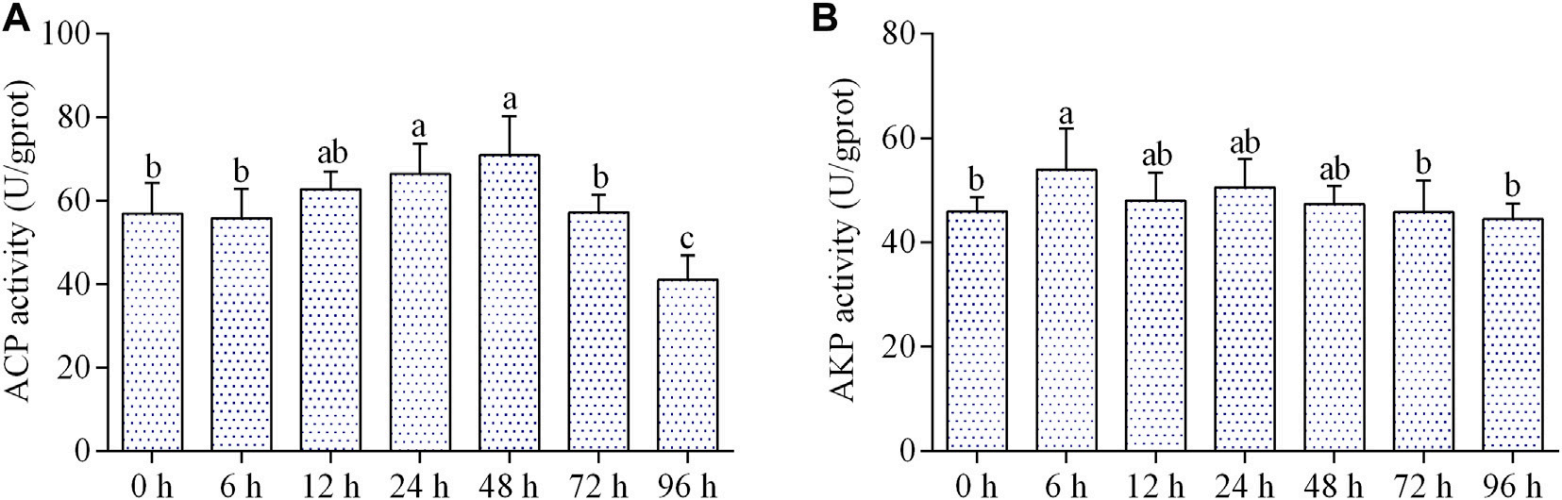
Effects of air exposure on immune parameters in hepatopancreas. **(A)** ACP activity; **(B)** AKP activity. Data were expressed as mean *n* ± SD (*n* = 5). Different letters indicated statistical significance (*p* < 0.05).

The ACP activity was significantly increased at 24 and 48 h of air exposure, and then significantly dropped at 96 h (Figure 4A). The activity of AKP increased markedly at 6 h of air exposure, and then maintained a control level from 12 to 96 h (Figure 4B).

### Effects of air exposure on transcriptomic

The transcriptome data were submitted to the SRA database (accession number: PRJNA817020). As shown in Table 1, a mean of 23,496,823 clean reads was obtained in all samples. The ranges of Q20 and Q30 were 98.16%–98.64% and 93.63%–94.94%, respectively. A total of 36,772 transcripts were assembled and 25,086 of them were annotated in six databases, including Uniprot, Pfam, GO, KEGG, KOG, and Nr (Table 2).

**TABLE 1:** Quality evaluation of transcription sequencing data.

Samples	Clean paired reads	Clean bases (G)	Q20 (%)	Q30 (%)	GC content (%)	Total mapped (%)
CL_1	24,633,480	7.12	98.23	93.81	39.92	73.8
CL_2	25,154,043	7.27	98.64	94.94	39.52	73.45
CL_3	25,610,559	7.4	98.3	94	40.27	71.47
AE_1	21,002,040	6.08	98.27	93.91	40.8	66.72
AE_2	20,368,416	5.94	98.16	93.63	40.83	70.45
AE_3	24,212,401	6.97	98.47	94.46	41.77	68.22
Average	23,496,823	7	98	94	41	71

**TABLE 2:** Summary of annotations of transcripts.

Category	Number of transcripts	Percentage (%)
Annotated in uniprot	22,894	62.26
Annotated in Pfam	22,295	60.63
Annotated in GO	17,377	47.28
Annotated in KEGG	15,529	42.23
Annotated in KOG	329	0.89
Annotated in Nr	22,939	62.38
Annotated transcripts	25,086	68.22
Total transcripts	36,772	

**FIGURE 5:**
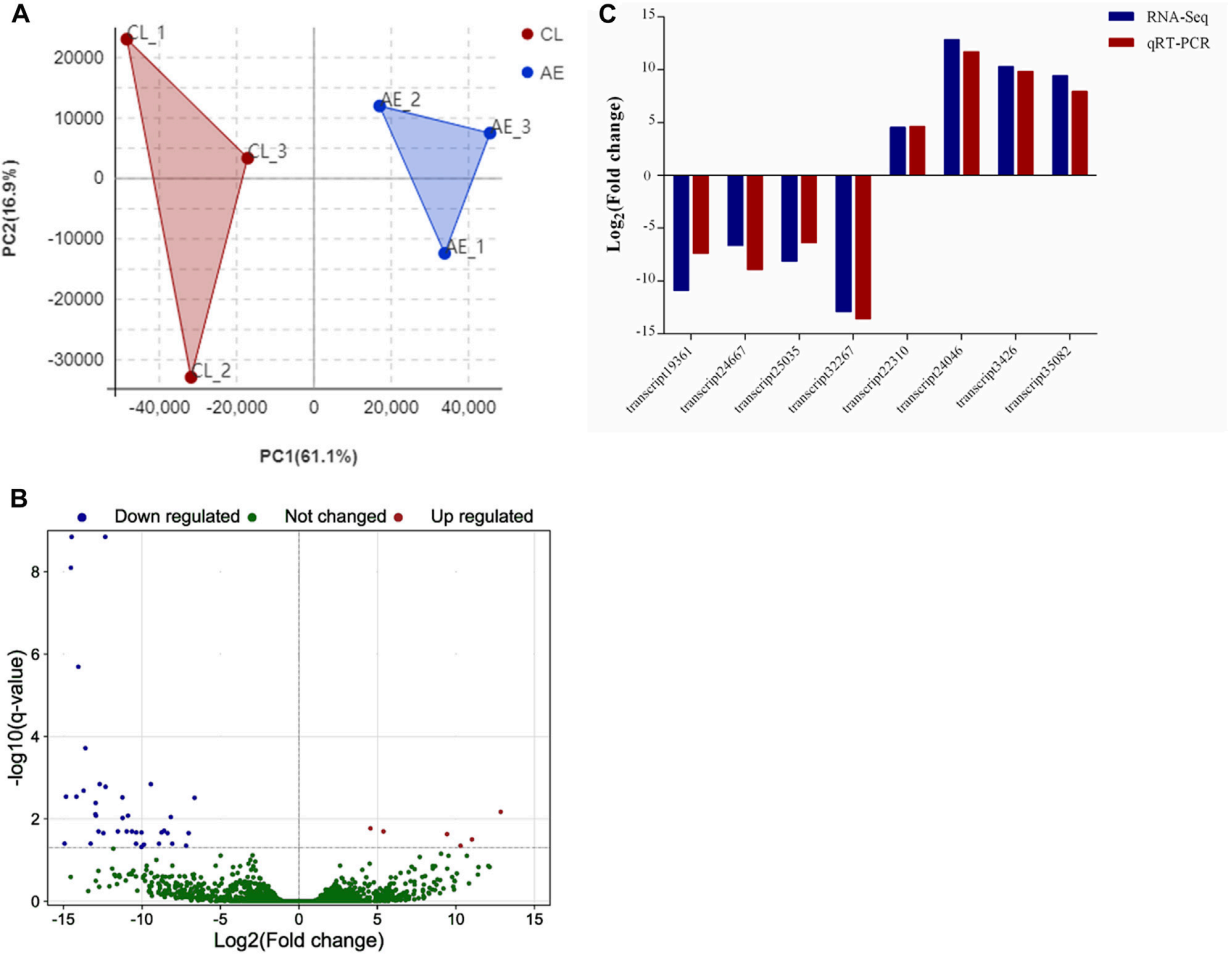
Identification and verification of DEGs. **(A)** PCA analysis of transcript expression in control (CL, 0 h) and air exposure groups (AE, 96 h); **(B)** Volcano map of DEGs; **(C)** Comparison of the relative fold change of RNA-Seq and qRT-PCR results.

As shown in Figure 5A, principal component analysis (PCA) of transcript expressions showed that samples from the control and exposure groups were clustered separately, indicating significant differences between the two groups. A total of 44 DEGs were identified in the hepatopancreas after 96 h of air exposure, including 6 upregulated genes and 38 downregulated genes (Figure 5B). To verify the results of transcriptome analysis, 8 transcripts were randomly selected to perform qRT-PCR. Figure 5C showed similar change tendencies from RNA-seq and qRT-PCR, suggesting the reliable results of RNA-seq.

**FIGURE 6:**
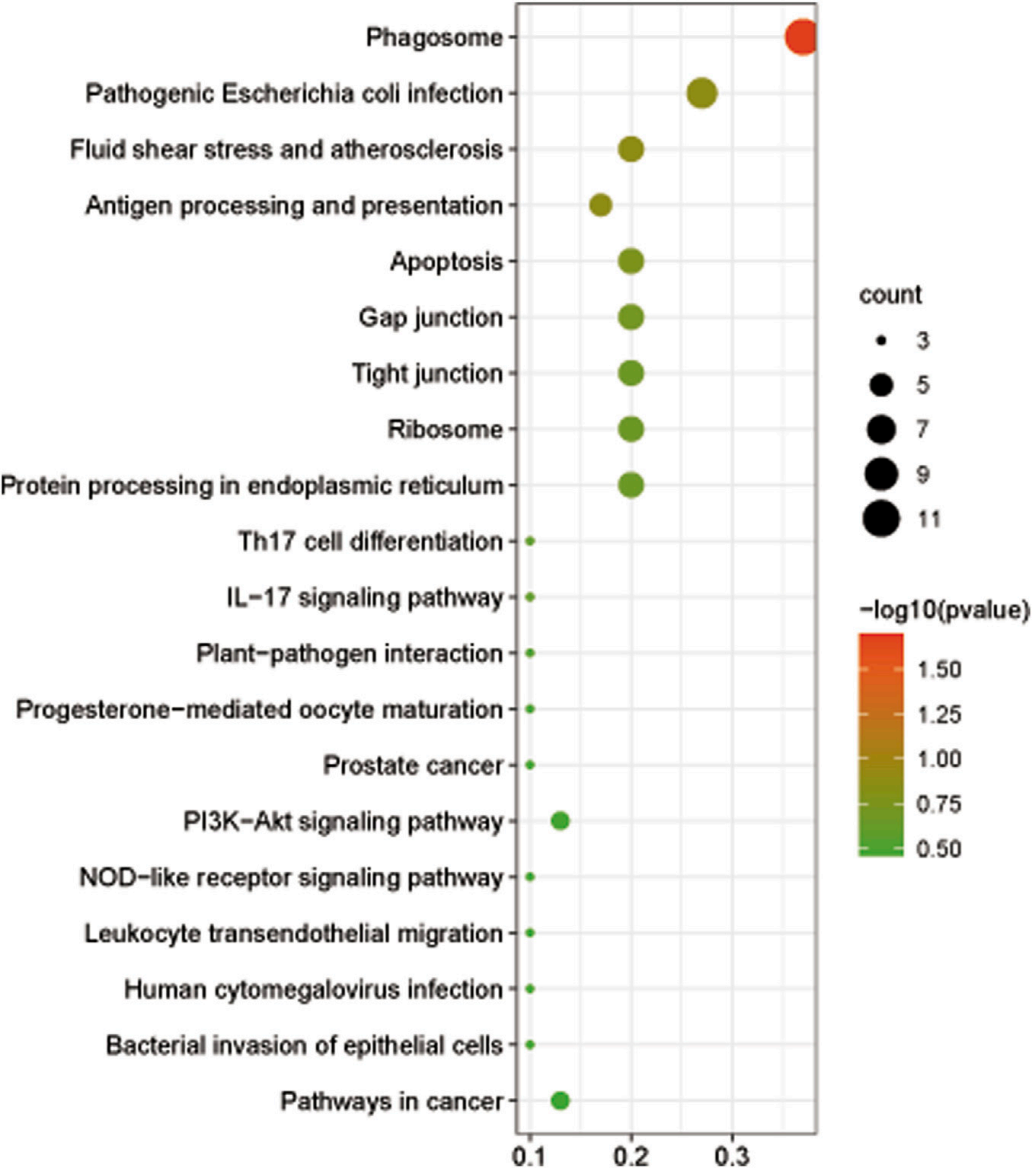
The bubble diagram showed the top 20 enriched pathways of DEGs at level 3.

The GO analysis was performed to understand the functional difference of DEGs. The results showed that DEGs were mainly enriched in the terms of intracellular non−membrane−bounded organelle and non−membrane−bounded organelle for cellular component ontology and the term of structural molecule activity for molecular function ontology (Supplementary Figure S1). Based on the KEGG pathways analysis, we found that DEGs were mainly enriched in pathways associated with the phagosome, pathogenic *Escherichia coli* infection, fluid shear stress and atherosclerosis, antigen processing and presentation, apoptosis, etc., (Figure 6). Eight DEGs involved in respiratory metabolism, immunity, and antioxidants were identified in this study and all of these genes were downregulated after 96-h air exposure (Table 3).

**TABLE 3:** List of 8 EDGs involved in glycolysis, TCA cycle, immune, and antioxidant defense.

Mechanism	Gene ID	Gene name	Log 2 (fold change)
Glycolysis	Transcript31400	Glyceraldehyde-3-phosphate dehydrogenase (*GAPDH*)	−10.4
TCA cycle	Transcript12039	Citrate synthase (CS)	−11.2
Immune	Transcript26259	Heat shock protein 90 alpha family class A member 1 (HSP90aa1)	−14.2
	Transcript32980	Heat shock protein 90 alpha family class B member 1 (HSP90ab1)	−8.59
	Transcript33919	Heat shock protein 90 alpha family class B member 1 (HSP90ab1)	−8.91
	Transcript30291	Ras-related C3 botulinum toxin substrate 1 protein (Rac1)	−8.07
	Transcript31256	Calreticulin (CLAR)	−10
Antioxidant defense	Transcript33245	Glutathione peroxidase 1 (GPx-1)	−7.03

## Discussions

Previous studies showed that air exposure can destroy the histological structures of many tissues, including gill, skin, intestines, and hepatopancreas, in aquatic animals (Lu et al., 2021; Harianto et al., 2022). In the current study, we observed the histopathological alterations of the hepatopancreas in *C. fluminea* under air exposure. We found that air exposure (6–96 h) caused the enlargement of the lumen and the vacuolation of the hepatopancreas. This finding indicated that air exposure induced tissue injury of the hepatopancreas in *C. fluminea*.

Respiration metabolism is the fundamental way for an organism to obtain energy. Aquatic animals usually perform aerobic respiration to provide energy for the organisms. However, when they undergo stress by adverse factors such as air exposure or hypoxia, they activate anaerobic respiration to adapt to the anoxic environment (Pihl et al., 1991; Ali and Nakamura, 2000). SDH is a key rate-limiting enzyme of aerobic respiration and is responsible for catalyzing the oxidation of succinic acid to fumaric acid and releasing ATP (Hägerhäll, 1997). In this study, the activity of SDH reduced from 24 h of air exposure. This may be due to that air exposure over 24-h resulted in cellular hypoxia in the hepatopancreas of *C. fluminum*, which suppressed electron transport chain and the rate of aerobic respiration metabolism. HK and PK are the two key rate-limiting enzymes of glycolysis. The former catalyzes glucose to glucose-6-phosphate and the latter catalyzes phosphoenolpyruvate to enolpyruvate (Cota-Ruiz et al., 2015). LDH is also a vital metabolism-regulating enzyme of anaerobic respiration and can catalyze pyruvate to lactic acid (Schurr and Payne, 2007). Previous studies have shown that the activities of LDH, HK, and PK can be altered by air exposure or hypoxia stress (Bao et al., 2019; Mattioli et al., 2019; Ding et al., 2020). In the present study, we found that the activities of HK, PK, and LDH increased first and then decreased during air exposure. Therefore, the increased activity of LDH in the 24 h indicated that anaerobic respiration in the hepatopancreas enhanced during early period of air exposure to provide additional energy for resistance to stress. However, the activity of LDH recovered to normal level during 48–96 h of air exposure. One reason might be that the structure and function of hepatopancreas of *C. fluminea* were impaired with prolonged air exposure, leading to inhibition of anaerobic respiration.

Under air exposure, aquatic animals may suffer hypoxia or anoxia stress, which will hinder the electron transport vector and promote the generation of ROS. When ROS exceeds the scavenging capacity of the antioxidant system, the disorder of the antioxidant system will occur, resulting in oxidative damage and even cell apoptosis of tissues (Pitkanen and Robinson, 1996). The effects of air exposure on the antioxidant system have been proven by growing evidence (Bao et al., 2019; Gao et al., 2021; Lu et al., 2021). SOD is one of the key enzymes in the antioxidant system and can catalyze superoxide ions into hydrogen peroxide (H_2_O_2_), which then is converted into water and oxygen by another antioxidant enzyme CAT (Martinez-Alvarez et al., 2005). GSH is an important non-enzymatic small molecule that exerts antioxidant effects by reacting with ROS or acting as a chain-breaker of free radical reactions (Xu et al., 2021). In this study, during 6–48 h of air exposure, SOD, CAT, and GSH content were increased, but recovered to normal or even decreased during 72–96 h. Furthermore, the content of MDA, a biomarker of oxidative lipid damage (Xu et al., 2020), was increased after 96 h of air exposure. These results demonstrated that short-term air exposure enhanced the antioxidant capacity of *C. fluminea*, but long-term air exposure caused lipid peroxidation damage in the hepatopancreas.

Like most invertebrates, the immune system of mollusks consists mainly of non-specific immunity that is sensitive to various environmental stresses, such as salinity shift and air exposure (Li et al., 2017; Zhang et al., 2021). ACP and AKP, two important hydrolases in lysosomes, are important in fighting disease and non-specific immune responses (Zhang et al., 2021). It has been reported that the activities of the two enzymes can be affected by air exposure in shellfish, such as *Pinctada fucata*, *Chlamys farreri*, and *Argopecten irradians* (Chen et al., 2007; Chi et al., 2017; Wei et al., 2021). In this study, we discovered that ACP and AKP activities were increased first (6–48 h) and then kept normal or even decreased during air exposure. Thus, our results suggested that at the early stage of air exposure, the non-specific immunity of *C. fluminea* was enhanced to resist stress, but the immune function was inhibited with the increase of exposure time.

Growing evidence has demonstrated that air exposure can induce changes in the expression of genes related to the glycolysis and tricarboxylic acid (TCA) cycle (Cheng et al., 2020; Zhou et al., 2021). In this study, the expressions of glyceraldehyde-3-phosphate dehydrogenase (GAPDH) and citrate synthase (*CS*) were downregulated after air exposure. GAPDH catalyzes the sixth step of glycolysis, converting glyceraldehyde 3-phosphate to D-glycolic acid 1, 3-diphosphate (Colell et al., 2009). CS catalyzes the condensation of oxaloacetic acid and acetyl-coA to produce citric acid, which is the key regulating step of the tricarboxylic acid (TCA) cycle (Roosterman and Cottrell, 2021). The downregulated expression of *GAPDH* and *CS* genes in this study indicated that 96 h of air exposure suppressed the glycolysis and TCA cycle of *C. fluminea.*

Heat shock proteins (HSPs) are a group of molecular chaperones and play important roles in various enverinemental stress responses (Junprung et al., 2017). Previous studies have shown that air exposure can influence the expressions of multiple subtypes of HSP genes, including *HSP70*, *HSP90*, and *HSP21*, in aquatic animals (Nie et al., 2020; Lu et al., 2021). In the current study, we found that the expressions of several *HSP90* genes (*HSP90aa1* and *HSP90ab1*) were downregulated after air exposure, revealing that HSP90 is closely related to the response of *C. fluminea* to air exposure. Glutathione peroxidase (Gpx) is a vital component in the antioxidant system and can reduce toxic peroxides to non-toxic hydroxyl compounds and promote the decomposition of H_2_O_2_ (Ighodaro and Akinloye, 2017). The expression of the *GPx* gene was upregulated by a short-term (8 h) air exposure (Lu et al., 2021). However, in this study, the expression of *GPx-1* was decreased after 96 h of air exposure, suggesting inhibited the antioxidant capacity of *C. fluminea*. Ras-related C3 botulinum toxin substrate 1 protein (Rac1) is Rho family small GTPase and exerts crucial roles in phagocytosis, inflammation, and immune response to extracellular stimuli (Feng et al., 2019). Calreticulin (CARL) is a calcium-binding chaperone that takes part in many immune processes, including phagocytosis and antigen presentation (Raghavan et al., 2013). In this work, the downregulated expressions of *Rac1* and *CARL* genes revealed suppressed immune function of *C. fluminea* by air exposure.

## Conclusion

Overall, this study clarified the effects of air exposure on the histology, physiology as well as transcriptome of the hepatopancreas of *C. fluminea* for the first time. Detection of physiological indicators revealed that the respiratory metabolism, antioxidant capacity, and immune function of *C. fluminea* were enhanced to resist adversity under a short time (0–48 h) of air exposure, while these physiological indices were inhibited under a longer time (72–96 h) of air exposure. RNA-seq identified 8 DEGs related to glycolysis, TCA cycle, immune, and antioxidant, and all of the 8 DEGs were downregulated after air exposure. These findings will contribute to explaining the coping mechanism of *C. fluminea* and other freshwater shellfish to air exposure.

## Data Availability

The datasets presented in this study can be found in online repositories. The names of the repository/repositories and accession number(s) can be found below: https://www.ncbi.nlm.nih.gov/, PRJNA817020.
