# Supplementary material for: Histological, physiological, and transcriptomic responses of hepatopancreas to air exposure in asian freshwater clam Corbicula fluminea
Source: Front Physiol. 2022 Aug 11;13:952744. doi: 10.3389/fphys.2022.952744 (PMC9402986; doi:10.3389/fphys.2022.952744)
Supplement: Supplementary file 1 [file Table1.DOCX]

# Supplementary Figures and Tables

**Supplementary Figures**


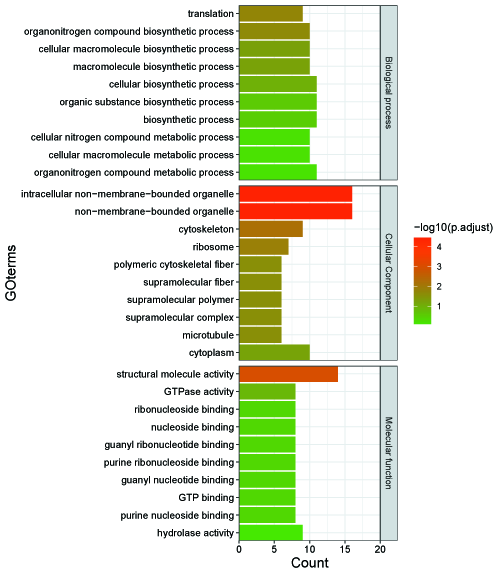


**Figure S1** The top 30 enriched GO terms of DEGs at level 2.

**Supplementary tables**

**Table S1.** Primer sequence used in qRT-PCR.

| Gene name | Primer sequence | |
| --- | --- | --- |
|  | Forward (5'-3') | Reverse (5'-3') |
| *EF1α* | TGCTGGTACTGGTGAGTTCG | CCATCCAGAGATTGGCACGA |
| *transcript19361* | CCAAACACGCTCAGACTCCC | AAACCATGAGTTGAAGGCGG |
| *transcript24667* | GAGGTGGCCAGTGAATGTGAA | AAAAAGGTGCCAAATGTGAAGAAC |
| *transcript25035* | ACATGACAAGCAGTTGACCGA | GCGCGCTTAATGAGCTATCC |
| *transcript32267* | TCGGACTATTTTAACTACAACATCG | CCGCCGTTTCTATTTTTGCT' |
| *transcript22310* | GCCCCAACCGCTACAAAATG | GTACCAGCCCAACAAGGACA |
| *transcript24046* | TGAATGTGGCTGTTGGTGGT | GGCGTCCTCTCCATTGTTCT |
| *transcript3246* | CGAGATCACTGACTCCGCCT | GCATTTCTTGCCCAGTAACGA |
| *transcript35082* | TTGGACGCTATCCTTTTCACG | TTCGCCTTTGACGATAGTGATGGTG |
